# The Mobile Lower Body Negative Pressure Gravity Suit for Long-Duration Spaceflight

**DOI:** 10.3389/fphys.2020.00977

**Published:** 2020-08-05

**Authors:** Neeki Ashari, Alan R. Hargens

**Affiliations:** ^1^Department of Orthopaedic Surgery, University of California, San Diego, San Diego, CA, United States; ^2^Department of Bioengineering, University of California, San Diego, San Diego, CA, United States

**Keywords:** lower body negative pressure, LBNP, ground reaction force, modeling, simulated gravity, artificial gravity, SANS, headward fluid shifts

## Abstract

Spaceflight Associated Neuro-ocular Syndrome, bone decalcification, and muscle atrophy are among the most prevalent risks associated with long-duration spaceflight. Implementing the lower body negative pressure (LBNP) method is a potential countermeasure for these risks. LBNP counteracts head-ward fluid shifts and generates ground-reaction forces (GRFs). GRFs are beneficial for maintaining bones and muscles by producing gravity-like loads experienced on Earth. Currently, LBNP devices are large/bulky, and usually require the subject to maintain a stationary position. However, our new mobile gravity suit is relatively small, untethered, and flexible in order to improve mobility in space. We hypothesized that this novel mobile gravity suit generates greater GRFs than a standard LBNP chamber. While lying supine, GRF data were recorded in both devices using foot sole sensors and a weight scale. At -40 mmHg, the gravity suit generated a mean maximum bodyweight of 125 ± 22% (*P* < 0.02) whereas the standard LBNP chamber generated 91 ± 24%. The standard LBNP chamber generated a single force on the stationary subject, which was expressed as A_W_(LBNP) = GRF, where A_w_ = cross-sectional area (CSA) of subject’s waist. However, the mobile gravity suit generated an additional force based on the following equation, (A_F_ + A_W_)LBNP = GRF, where A_F_ = CSA of subject’s feet. The additional force was further expressed as F1 + F2 = A_F_ × LBNP, where F1 = spinal loading force, F2 = waist shear force, and A_F_ × LBNP = the total downward foot force. Thus, the mobile gravity suit produces higher percentages of bodyweight due to the suit’s novel design.

## Introduction

Spaceflight Associated Neuro-ocular Syndrome (SANS), previously known as Visual Impairment Intracranial Pressure (VIIP), is a major risk associated with long-duration spaceflight. During prolonged missions, optic disk edema, posterior globe flattening, decreased near vision, and hyperopic shifts are hallmarks of SANS (Zhang and Hargens, 2018). This risk stems from the lack of gravity, which causes a headward shift of blood and other body fluids (Sater et al., 2020). As a result, astronauts experience a mild, but constant elevation of intracranial pressure (ICP) unlike alterations of ICP with posture on Earth. Although SANS is a recently-identified critical risk of spaceflight, it is not the only physiological adversity astronauts may endure. Long-term microgravity exposure is also responsible for the reduction of mechanical loads, which reduce bone density, and muscle force generation (Akima et al., 2003). Furthermore, an astronaut’s movement between modules, aerobic activity, and extra-vehicular activity components are amongst the leading causes of musculoskeletal injuries (Scheuring et al., 2009). This becomes a major concern as astronauts return from space to weight-bearing environments, such as Earth or even potentially Mars. On Earth, gravity is responsible for supplying resistance in our everyday life (Kohrt et al., 2009). Most commonly, we experience resistance through the ground-reaction forces (GRFs) our bodyweight generates underneath our feet. GRFs are critical forces that help increase bone growth and maintain muscle structure and function (Boda et al., 2000; Witt and Ploutz-Snyder, 2014). In order to minimize musculoskeletal loss and injuries, it is essential to develop effective techniques that reproduce gravitational forces for microgravity conditions.

Ensuring mechanical loads on the human body is an essential necessity for long-duration spaceflight missions. Studies show that bearing the mechanical load of your bodyweight serves as a fundamental stimulus for maintaining musculoskeletal health. In microgravity conditions, there is a lack of external forces, which inhibits bone tissue from experiencing changes in strain energy – an important fluctuation we experience on Earth (Vico and Hargens, 2018). Without these changes, bones become more prone to breaks and fractures (Dadwal et al., 2019). Currently, the International Space Station (ISS) incorporates exercise regimens to simulate artificial gravity to generate GRFs. Unfortunately, treadmills generate only a fraction of the GRFs compared to those generated on Earth (Cavanaugh et al., 2010). Studies show that walking, running, and squatting in space generates a reduced GRF by 77, 75, and 65% (Cavanaugh et al., 2010). However, the advanced resistive exercise device (aRED) is an actively used countermeasure device in the ISS. Through its dynamic characteristics, it can simulate inertial loading up to 2,675N (Vico and Hargens, 2018; Sibonga et al., 2019). Studies show that consistent aRED usage maintains bone density and increases bone renewal (Smith et al., 2008). However, remaining stationary in exercise devices for 1–2 h per day sacrifices critical crew time for operational and science-related tasks.

A common technique to potentially alleviate musculoskeletal and head-ward fluid shift issues is applying lower body negative pressure (LBNP). LBNP induces a blood shift from upper body to lower body compartments to partially reverse cephalic fluid shifts that occur during weightlessness (Goswami et al., 2018). Additionally, LBNP produces reflexive hemodynamic and cardiovascular control responses similar to when experiencing an increased gravitational load (Goswami et al., 2018). This vacuum-style technique (below ambient pressure) applies a gravitational-like stress onto the cardiovascular system and generates GRFs beneath the feet to simulate axial loading. By increasing foot-ward loading through LBNP, this may have applications for orthopedic rehabilitation and spaceflight deconditioning (Goswami et al., 2018). These gravitational-like factors are imperative for maintaining bone density and muscle generation (Kohrt et al., 2009; Vico and Hargens, 2018). Generally, LBNP devices come in the form of a horizontal chamber.

A standard LBNP chamber is extremely heavy and bulky. Thus, it is excluded from any in-flight missions to the ISS or beyond Earth orbit. Due to the large volume of the chamber, it requires more power consumption when generating stronger pressures. Additionally, the chamber is completely static and requires the user to remain inside for extended periods of time. Currently, the Roscosmos (Russian Space Agency) has its own LBNP countermeasure device in the ISS, called the *Chibis* (Yarmanova et al., 2015). This countermeasure device has no mobility, requiring the user to always be connected to a stationary vacuum and wall-mounted power supply (Yarmanova et al., 2015). Nearing the end of their flight missions, cosmonauts use the *Chibis* to apply a stress onto their cardiovascular system. This prepares their heart to feel similar stresses upon their return to Earth’s gravity. Lastly, none of these iterations feature a safe, comfortable, and mobile solution.

**FIGURE 1:**
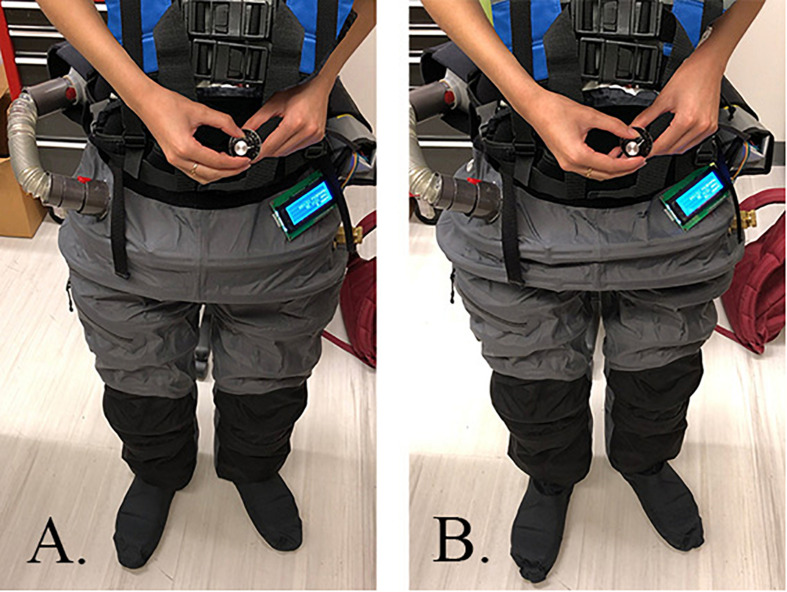
**(A)** Mobile gravity suit without negative pressure activation and **(B)** mobile gravity suit with negative pressure activation (−10 mmHg).

**FIGURE 2:**
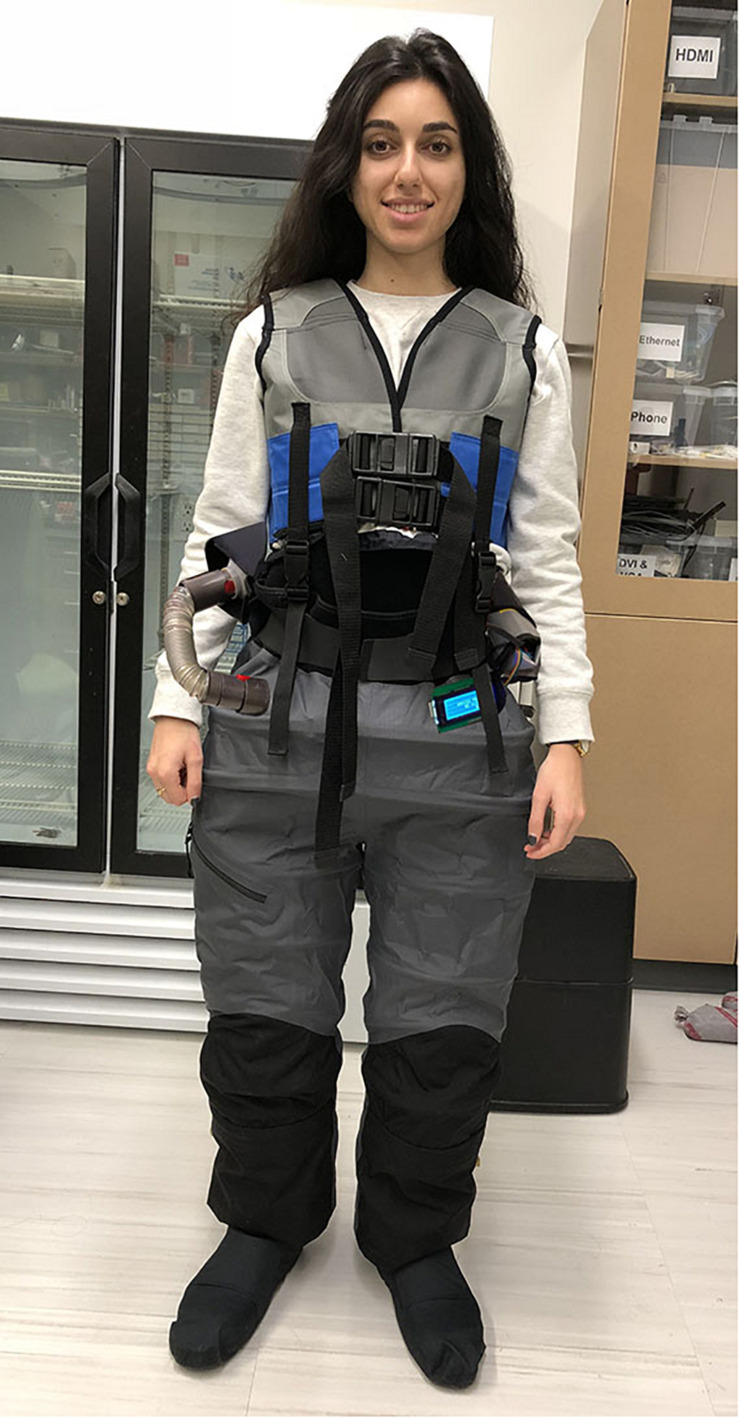
Full body view of the mobile gravity suit.

However, we designed and developed a new LBNP device in the form of wearable trousers – called the mobile gravity suit (Figures 1A,B, 2). The mobile gravity suit is a small, untethered, and flexible intravehicular activity (IVA) suit. This trouser-like suit is designed for astronauts to comfortably wear and begin applying the LBNP technique without reducing crew time. The negative pressure is generated by its own portable vacuum system, ensuring full mobility, and user-control. Additionally, the gravity suit’s endoskeleton is equipped with its own pressure/thermal control system and three safety features. Due to the gravity suit’s biomechanical design, the flexible exoskeletal membrane axially contracts under negative pressure. This mechanical and dynamic characteristic may provide an additional force that the static LBNP chamber does not generate.

Thus, we hypothesized that our new mobile gravity suit generates greater GRFs than a standard LBNP chamber. In this study, we compare the two devices’ experimental GRF data and explore how their different designs affect that relationship. Additionally, this paper will detail the biomechanics behind the mobile gravity suit and its physiological advantages.

## Materials and Methods

### Approval and Recruitment

This study was approved by the Institutional Review Board of the University of California, San Diego. Each subject read the consent form and provided informed, written consent. Our previous study collected gravity suit GRF data from eight healthy subjects (6 females and 2 males) with an average age ± SD: 24 ± 6 years, average height ± SD: 168 ± 6 cm, and average weight ± SD: 57 ± 8 kg (Petersen et al., 2019). As for the LBNP chamber GRF data, we recruited a total of six healthy subjects (3 males and 3 females) with an average age ± SD: 23.3 ± 4.3 years, average height ± SD: 170 ± 7 cm, and average weight ± SD: 64.4 ± 12.7 kg.

### The Gravity Suit and Model

We designed and developed the mobile gravity suit in the form of wearable trousers that is fully equipped with its own portable vacuum system, pressure and thermal control system, safety shut-off system, and spinal loading system. The suit’s exoskeletal membrane envelops the user from the waist down, encapsulating the feet. This membrane consisted of an airtight yet breathable Hyprotex fabric. This ensured pressure retention, all while maintaining the suit’s temperature and humidity. The endoskeleton of the suit was structured using 1/4″ cross-linked polyethylene (PEX) tubing. This internal support structure was composed of repeated ring-shaped PEX tubing, which maintained several inches of clearance between the user and the suit. Each ring was placed into a double or triple tier ring structure to prevent warping and/or deformation under negative pressure. The ring sets were spaced evenly throughout the suit to maintain a flexible user environment and to promote accordion-like axial contraction. This novel biomechanical design ensured an additional dynamic force that a static LBNP box did not have.

The knee joint was designed to ensure zero skin contact at both static and dynamic positions. The knee joint employed a “pac-man” open-mouth shape, allowing for free space at the knee anterior. The knee joint was developed using PEX tubing. To ensure extra reinforcement of each ring-stack placement, a strong adhesive fabric was developed. The fabric was then layered over every square-inch of the endoskeleton, tightly retaining all ring-stacks. This detail provided esthetic yet smooth surface properties for dynamic air-flow.

At the waist of the suit, 3/8″ PEX tubes were used to develop two separate two-tier stacked rings to form the aperture. This aperture support structure used larger diameter rings that extend about 3–4 inches outward below the user’s iliac crest. This design ensures dynamic air-flow and less strain on the user’s cardiovascular system. Above the aperture, a vacuum docking port was placed. The docking port was fabricated/modified out of a Dyson accessory female counterpart, allowing it to match the hose’s male counterpart accessory. Together, they would fully “click” into place. In the event of an emergency, the user would have free-control to unclick the hose to ensure immediate relief of the entire system. This also served as an additional safety feature (manual).

The suit was also equipped with a customized portable vacuum system. The portable vacuum was developed out of a 90 mm 12-blade metal-ducted brushless fan. The 22.2V fan motor was supported by a Lectron Pro 22.2V 5200 mAh LiPo battery and an electronic speed controller. Together, they produced approximately 80,000 RPMs and 3,620 grams of thrust to generate a strong negative pressure. The voltage of the vacuum was scaled with a portable variable resistor controlled by the user. The vacuum itself was housed in a 3D-printed enclosed CAD casing designated on the user’s right hip.

Near the suit’s aperture, a one-way 3/4″ mechanical pressure relief check-valve was installed to allow air flow in when reaching a negative pressure threshold of −50 mmHg (cut-off dosage). This mechanical safety feature ensured immediate leakage in the event the suit reaches a dangerous negative pressure threshold for the user. While −50 mmHg is a suggested cut-off dosage, it is certainly not the standard. During the static experiment, this cut-off dosage was selected to avoid discomfort and knee buckling among subjects. Located inside the suit is a pressure, temperature, and humidity sensor. Each autonomous sensor was housed together inside an internal safety pouch. Once activated, the sensors relayed information via Bluetooth to the LCD screen’s Arduino mega. This then displayed a digital output of pressure (mmHg), temperature (C), and humidity (%) on the LCD screen. The LCD screen was designated on top of the suit’s aperture, which provided an aerial view for the user. As an electrical safety feature, the pressure sensor’s Arduino nano incorporated a vacuum shut-off algorithm if the suit ever reached a dangerous negative pressure threshold. Additionally, each ankle was equipped with a one-way 1/4″ brass pressure relief check-valve to achieve minimal air flow in. Each check valve employed a reverse ball spring mechanism. As the suit generated negative pressure (via portable vacuum), it pulled the ball back against the spring and uncovered the inlet hole – allowing atmospheric air flow into the suit. The check-valve is calibrated by sensitivity. Thus, it was calibrated to open at around −15 mmHg. Since the inlet of the check-valve is so small (1/4″), it only allowed for minimal air flow in. Minimal air flow in allowed us to regulate temperature and humidity inside the suit when engaging in dynamics or statics.

A spinal loading vest was added to connect to the waist of the suit. The shoulder pads employed an even distribution area. This equalized the applied mechanical load onto the user’s shoulders and spine to simulate the diurnal changes we experience on Earth. Lastly, we implemented Crocs shoes to prevent compression at the bottom of the suit. Crocs provided a rigid, yet durable structure around the feet. Overall, the completed gravity suit is shown without negative pressure activation in Figure 1A, with negative pressure activation (−10 mmHg) in Figure 1B, and a full body view in Figure 2.

A static and force-balance analysis was conducted to target the applied and residual resulting forces on the device. Through this model, we could predict the GRFs generated under each individual. This was expressed as:

$$\left({\text{A}}_{\text{F}}+{\text{A}}_{\text{W}}\right)\,\text{LBNP}=\text{GRF}$$

Where *A_*w*_* = cross-sectional area (CSA) of the subject’s waist. The additional force could be further expressed as:

$$\mathrm{F1}+\text{F}\,2={\text{A}}_{\text{F}}\times \text{LBNP}$$

Where *F1* = spinal loading force, *F2* = waist shear force, and *A_F_ × LBNP* is the total downward reaction foot force during axial contraction.

### LBNP Chamber and Model

The LBNP Chamber was designed and manufactured at the Scripps Institution of Oceanography Machine Shop at UC San Diego. This four-sided static chamber was built with 1-inch thick Plexiglas to sustain high negative pressures. The front panel incorporated a 182.8 cm circumferential elliptical aperture. In most cases, the aperture left about 9-inches of clearance between itself and the user. Around the aperture was a flexible neoprene waist to ensure minimal leakage. Above the aperture, a raised steel-beam was placed to support the friction-less backboard (Cavanaugh et al., 1992). To support the user when lying supine, leg, thigh, and hip bungee cord slings were installed inside the chamber.

The original force model for this device can be expressed as (Hargens et al., 1991; Boda et al., 2000):

$${\text{A}}_{\text{W}}\times \text{LBNP}=\text{GRF}$$

Where *A_*w*_* = CSA of the subject’s waist.

### Experimental Design

The gravity suit was suspended inside of the LBNP chamber in order to utilize a friction-less ground-based analog. This ensured more accurate GRF data from the suit, as there will be less friction against the subject’s back. As the subject would don the suit, their legs, thighs, and hips would be suspended using the LBNP chamber’s bungee cord slings. The suit’s negative pressure system was activated from zero to 40 mmHg of negative pressure, using 10 mmHg intervals. Each interval was roughly 15-s. At each interval, the force was recorded using Tekscan Foot Sole Sensors.

For the LBNP chamber, each subject was instructed to lie supine. Their legs, thighs, and hips were suspended with bungee cord slings. Their back was supported with a non-resistive backboard sling. A neoprene seal enveloped the subject’s waist, maintaining a tight seal. All subjects were exposed to negative pressures from zero to 40 mmHg, using 10 mmHg intervals. Each interval was roughly 15-s. At each interval, the force generated onto the scale was recorded.

### Measurements

Tekscan Foot Sole sensors were placed inside each sole of the gravity suit’s shoes. Each sensor was graded with loaded cells to provide distributed force mapping underneath the subject’s foot. This was then quantified into GRFs. However, the LBNP chamber used a calibrated digital scale that was vertically mounted inside the panel door, while the gravity suit employed TekScan sensors beneath the feet.

### Statistics

The means ± standard deviations for the gravity suit GRF were compared to the standard LBNP chamber. A two-tailed *t*-test was used to compare the two conditions to determine statistical significance for each average percent bodyweight generated. This was done by comparing the normalized weights of the gravity suit trials with the normalized weights of the LBNP chamber trials, demonstrating a significant difference (set at *P* < 0.05) in the two conditions for each pressure interval. A correction for multiple comparisons adjusting for the total number of statistical tests was not performed because the *t*-tests were planned before they were conducted. From the fundamental analysis described above in the methods, we arrive at the theoretical expression for the GRF, which is (A_F_ + A_W_)LBNP = GRF. As we see from the expression, the GRF is linearly related to the independent variable LBNP, and thus a linear regression was an appropriate choice to fit the data and to obtain the relevant coefficients.

## Results

All subjects who participated in these studies produced reliable data and showed no pre-syncopal symptoms.

Following the gravity suit’s GRF protocol, subjects generated a mean GRF of 0%, 13 ± 3%, 41 ± 5%, 75 ± 11%, and 125 ± 22% of their total bodyweight at 0, −10, −20, −30, and −40 mmHg, respectively. Observational results displayed that subjects generated a comfortable 90° knee flexion at −20 mmHg, while still generating approximately 41% of their total bodyweight. At −20 mmHg for *N* = 1, a subject generated a temperature and humidity of 23 ± 1°C; 47 ± 3%, respectively, inside the suit (Petersen et al., 2019). The gravity suit’s maximum GRF increase was 25% higher relative to one bodyweight when standing upright. In order to generate about one bodyweight in the gravity suit, users had to implement approximately −35 mmHg.

**FIGURE 3:**
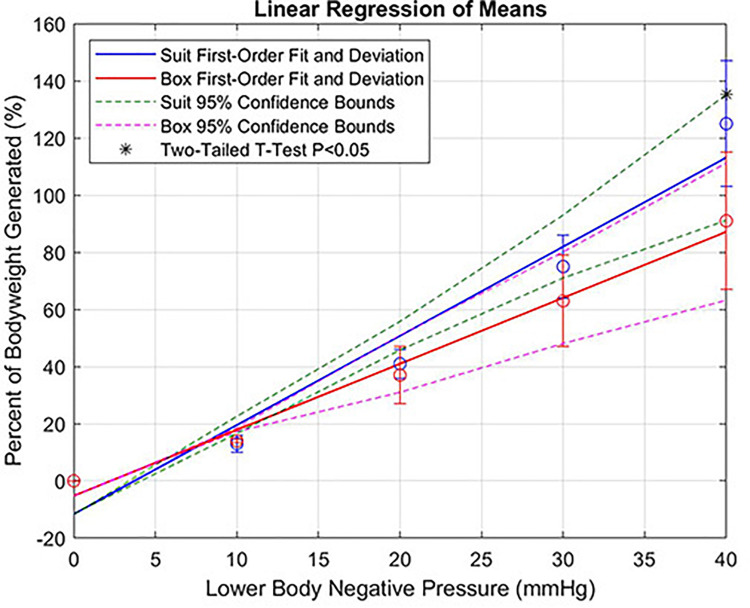
Percent of bodyweight generated (gravity suit vs LBNP chamber).

Following the LBNP chamber’s GRF protocol, subjects generated a mean GRF of 0%, 15 ± 0.66%, 37 ± 10%, 63 ± 13%, and 91 ± 24% of their total bodyweight at 0, −10, −20, −30, and −40 mmHg, respectively. In order to generate about one bodyweight in the LBNP chamber, users had to implement at least −45 mmHg. This high negative pressure threshold was −10 mmHg greater than that the gravity suit’s negative pressure generation. Each pressure stage (−10, −20, −30, and −40 mmHg) underwent a *t*-test and provided *p*-values less than 0.40, 0.40, 0.07, and 0.02, respectively. Compared to the standard LBNP chamber, the gravity suit’s mean bodyweight generation at maximum pressure (−40 mmHg) increased by 37% with a statistically significant *t*-test (*P* < 0.02). A previous study that used a larger pool of subjects showed that it takes around −100 mmHg to generate a single bodyweight (Hargens et al., 1991). However, the gravity suit required substantially less to generate a single bodyweight. A linear order regression of means between the Gravity Suit vs the LBNP Chamber can be seen in Figure 3.

**FIGURE 4:**
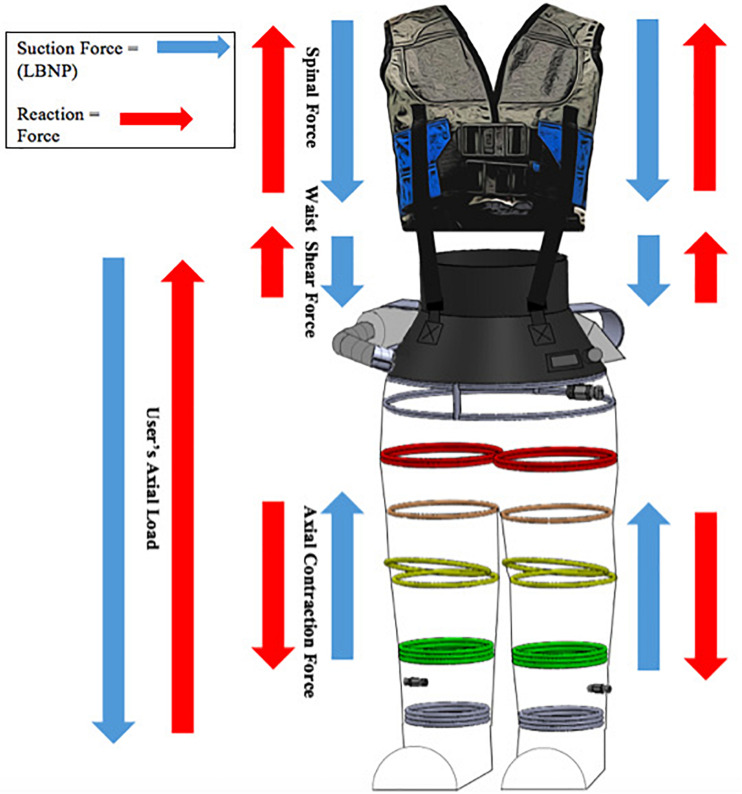
Mobile gravity suit force balance analysis.

The gravity suit’s force balance analysis (FBA) illustrated where the resulting reaction forces are derived. Upon vacuum activation, a downward force was generated at the spinal loading vest’s shoulders and a downward force was generated at the flexible neoprene waist. Since the loading vest and neoprene waist seal were connected to each other, they supplied a downward force in series. In result, they produced an upward reaction force. At the same time of this occurrence, the bottom of the suit began to axially contract, supplying an upward force underneath the subject’s feet. In result, the subject’s feet counteracted the axial contraction, therefore supplying a downward reaction force. The behavior of both occurrences was compared to an accordion-like mechanism. The gravity suit’s FBA in Figure 4 shows an additional reaction force that the standard LBNP chamber does not have.

**FIGURE 5:**
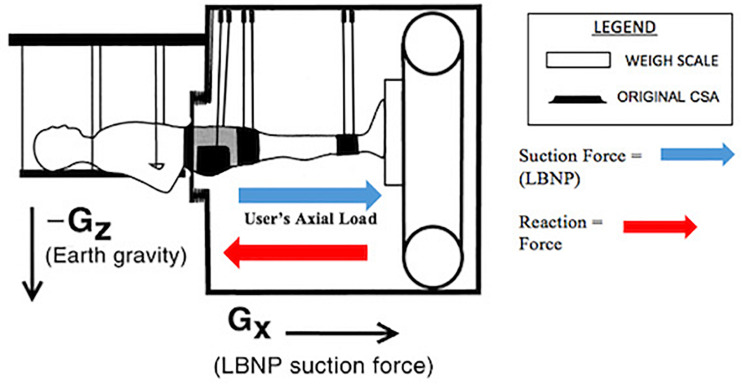
LBNP chamber force balance analysis.

The LBNP chamber’s FBA also illustrated where the resulting reaction forces were derived. The subject’s axial load supplied a downward force, which produced an upward reaction force. The LBNP chamber FBA is depicted in Figure 5.

## Discussion

### Gravity Suit vs LBNP Chamber

The primary findings of this study support our hypothesis that the gravity suit generates greater GRFs than the standard LBNP chamber. The suit’s results show a significantly higher mean maximum GRF of 125 ± 22% (*P* < 0.02) of their total bodyweight in comparison to the chamber’s mean maximum GRF of 91 ± 24% of their total bodyweight. The data show that as the negative pressure increases, the GRF difference between the gravity suit and LBNP chamber also increases. Therefore, with respect to our pressure intervals, higher levels of negative pressure lead to a significant *p*-value (*P* < 0.02). Furthermore, increasing negative pressure beyond −50 mmHg or even just by increasing the sample size, may yield more significant *p*-values. Additionally, the gravity suit requires −10 mmHg less to generate a single bodyweight in comparison to the standard LBNP chamber. This is beneficial as lower LBNP levels apply less stress to the crew member’s cardiovascular system, in comparison to higher levels of LBNP.

We suggest the gravity suit’s novel physiological and biomechanical design may be a primary reason for this GRF increase and minimal negative pressure usage. Since the suit’s flexible endoskeleton is composed of a repeated ring-shaped PEX tubing, this allows the structure of the suit to axially contract under negative pressure (Figure 4). This behavior is analogous to an accordion-like mechanism, extending and curtailing (Figures 1A,B). Furthermore, the shoe structure serves as a rigid platform underneath the user’s feet. This dynamic feature supplies an additional force that the rigid LBNP chamber does not have. The LBNP chamber’s robust yet strictly rigid structure only allows for one force in comparison to the gravity suit’s dual force dynamic feature.

Additionally, the gravity suit’s aperture ensures 3–4 inches of minimal clearance between its user in comparison to the LBNP chamber’s 9 inches of clearance. This may affect the waist shear force for each device. Under negative pressure, the flexible neoprene waist seal around each aperture inverts. As it inverts, it supplies a load onto the device and user. Since the gravity suit has a smaller area of clearance, we approximate 1/4 of the suction force is applied to the gravity suit, while 3/4 of the suction force are applied to the user contributing to their higher GRF generation. However, the standard LBNP chamber has a much larger aperture clearance. Thus, we approximate that ½ of its suction force is applied onto the LBNP chamber, while the other ½ is applied to the user contributing to the their minimal GRF generation. Overall, the gravity suit’s flexibility and smaller aperture clearance supplies maximum axial force contraction without limitations. However, the LBNP chamber’s larger aperture sustains horizontal tugging and resistance due to the large elliptical diameter and rigid properties. This can limit the amount suction force applied to the user.

### The Gravity Suit Force Model

The force model is as follows, (A_F_ + A_W_)LBNP = GRF, where A_F_ = cross-section area of feet and A_W_ = CSA of waist. Since the gravity suit is also equipped with a spinal loading vest, which is attached to the neoprene waist seal, their mechanical loads work in series. Thus, the force model is further expressed as: F1 + F2 = A_F_(LBNP), where F1 = spinal loading force, and F2 = waist shear force. In equivalence, the bottom of the suit’s exoskeletal membrane axially contracts upwards causing a downward foot force, hence A_F_(LBNP). This additional force supports the gravity suit’s results for generating a stronger force than the LBNP chamber.

### LBNP Chamber Force Model

According to a previous study conducted on the LBNP chamber, a force model was developed through an FBA (Boda et al., 2000). Since the LBNP chamber in our study does not implement a spinal loading vest and/or dynamical material properties, it only generates a single resultant force in comparison to the gravity suit dynamic force feature. The force model for the LBNP chamber is as follows, A_W_(LBNP) = GRF, where A_W_ = CSA of waist.

### Limitations

Limitations for the gravity suit include restricted parameter sizes due to the suit’s tailored volume. Due to the suit’s parameter constraints, primarily females with specific waist and height parameters were selected for participation. This caused a shortage of subjects, and thus a smaller data set. Like extravehicular activity suits, IVA suits (the gravity suit) follow the same rules in terms of anatomical fit. If not, the suit’s biomechanical movement is hindered. For example, if the knee joint does not align with the user’s knee joint then obstruction against the knee will occur. With respect to astronautic use, we will collect each astronaut’s biometrics and use that data when developing the suit. For commercial (high volume) use, we would make five different sizes (XS, SM, M, L, and XL) with sizing charts to explain the biometrics of each.

## Conclusion

Overall, the gravity suit serves as a user-driven and mobile countermeasure that may maintain cardiovascular, visual, and musculoskeletal health without sacrificing crew time. The data show that the gravity suit generated greater GRFs than a standard LBNP chamber. As a result, the gravity suit generated a 37% greater mean maximum bodyweight (*P* < 0.02). This substantial increase allows astronauts to enhance their mechanical loading and resistance exercises. Regardless of statistical significance, the gravity suit alone is still far more advantageous than the standard LBNP chamber. The gravity suit ensures mobility, flexibility, and safety for the comfort of each user, while the standard LBNP chamber is large, immobile, and too bulky for spaceflight. With the gravity suit, astronauts will be able to float freely around the space station while adhering to their every day tasks. However, this device is not just relevant for astronauts. Once space travel becomes commercialized, this device may ensure the health of future civilian space travelers. It is important to develop effective devices, like the mobile gravity suit, that simulate the very conditions our bodies on Earth depend on. This innovation may be pivotal for the journey to Mars. In summary, by comparisons to a standard LBNP chamber, the mobile gravity suit provides higher GRFs on a safe and fully mobile scale. Due to the gravity suit’s smaller volume and biomechanical design, it requires less negative pressure to achieve a given GRF.

## Data Availability Statement

All datasets presented in this study are included in the article/supplementary material.

## Ethics Statement

The studies involving human participants were reviewed and approved by UCSD Human Research Protections Program Office. The patients/participants provided their written informed consent to participate in this study. Written informed consent was obtained from the individual(s) for the publication of any potentially identifiable images or data included in this manuscript.

## Author Contributions

NA designed and developed the mobile lower body negative pressure gravity suit, performed the experiments, analyzed and interpreted the results, developed the gravity suit’s mathematical model, wrote the manuscript, and prepared the figures. NA and AH designed and discussed the study and experimental design and obtained IRB approvals. AH revised the manuscript. Both authors read the manuscript for submission.

## Conflict of Interest

The authors declare that the research was conducted in the absence of any commercial or financial relationships that could be construed as a potential conflict of interest.

## Abbreviations

aRED: Advanced Resistive Exercise Device
GRF: Ground-Reaction Force
IVA: Intravehicular Activity
LBNP: Lower Body Negative Pressure
SANS: Spaceflight Associated Neuro-ocular Syndrome
VIIP: Visual Impairment Intracranial Pressure.
